# Moving Policy Toward a Whole School, Whole Community, Whole Child Approach to Support Children Who Have Experienced Trauma

**DOI:** 10.1111/josh.12957

**Published:** 2020-11-12

**Authors:** Deborah Temkin, Kristen Harper, Brandon Stratford, Vanessa Sacks, Yosmary Rodriguez, Jessica D. Bartlett

**Affiliations:** ^1^ Child Trends, 7315 Wisconsin Avenue, Ste 1200W Bethesda MD 20814; ^2^ Child Trends, 460 Totten Pond Road, Suite 260 Waltham MA 02451

**Keywords:** trauma, school health, school health policy, education policy, adverse childhood experiences, WSCC model

## Abstract

**BACKGROUND:**

As attention to the potential negative outcomes of childhood trauma has grown, so have calls for schools to take an active role in supporting students experiencing trauma. These calls extend beyond efforts initiated by individual schools to include those mandated by state law, which largely focus on teacher training and on screening for adversity.

**METHODS:**

This article explores the evidence base and limitations for current approaches in state law and explores how policies to address other student health, safety, and wellness issues can help either ameliorate or exacerbate students' experiences with trauma.

**RESULTS:**

Few trainings for nonclinical staff have rigorous evidence of effectiveness, and based on evidence of teacher trainings on other topics, cannot work in environments that do not actively reinforce and encourage the application of that knowledge. Trainings also largely do not acknowledge the structures and systems, including systemic racism within schools, that may contribute to disparate rates of adversity for black and American Indian and Alaskan Native children. Screening carries several risks, including confounding adversity with experiencing trauma, missing broader contextual adversity, and potentially retraumatizing children.

**CONCLUSIONS:**

State policymakers need to take a more holistic approach in creating policies to support students experiencing trauma.

A growing research base demonstrates a strong association between the experience of trauma in childhood and poor physical health, mental health, and academic functioning, among other long‐term negative outcomes.^1^, ^2^, ^3^, ^4^, ^5^, ^6^ Childhood trauma is the experience of an actual or threatened harmful event or set of circumstances that leads to emotional pain and overwhelms the child's ability to cope.^7^, ^8^, ^9^, ^10^ The prevalence of trauma varies by type of event or circumstance (eg, child abuse and neglect; intimate partner violence; extreme poverty; bullying; parental incarceration; discrimination; natural disasters; and pandemics) and method of measurement, but ranges between 4% and 71%.^11^, ^12^, ^13^

As attention to childhood trauma has grown, so have calls for schools to take an active role in supporting students experiencing trauma.^14^, ^15^ These calls extend beyond the efforts of individual schools to those mandated by state law.^16^ As of 2017, laws in at least 11 states encourage or require schools to train staff about trauma^16^ and this number continues to grow.^17^ Several states also recommend or require the use of screening tools to identify students who may be experiencing trauma.^18^ Unfortunately, even as policymakers require schools to implement these practices, there is limited evidence as to their effectiveness.^19^

The current policy approach for addressing trauma in schools focuses narrowly on identifying and responding to the issue. According to the Substance Abuse and Mental Health Services Administration (SAMHSA), trauma‐informed systems must not only *recognize* trauma and *respond* with appropriate supports, they must also *realize* the role of trauma in contributing to other behaviors and needs, and actively work to avoid *retraumatization*.^20^ Without grounding trauma within the broader health needs of students, and recognizing how trauma is both affected and ameliorated by how those needs are addressed, current policy strategies may fail to create the supportive school contexts necessary for students to thrive.

The Whole School, Whole Community, Whole Child (WSCC) framework illustrates how all aspects of a school's approach to mental, physical, and social health work together, whereby policies, processes, and practices are integrated across these issues to promote optimal conditions for improving health and learning.^21^ The WSCC captures schools' efforts across 10 domains including nutrition, physical education, health services, counseling, psychological and social services, social and emotional climate, physical environment, employee wellness, family engagement, and community involvement to promote safety and health so that students can be engaged, challenged, and supported in learning. In this article, we examine the school‐based strategies currently encouraged in state laws addressing childhood trauma, identify gaps in these strategies and their evidence base, and propose a new state policy framework to support schools in taking a more holistic trauma‐informed approach.

## LITERATURE REVIEW

### Childhood Adversity, Adverse Childhood Experiences, and Trauma

To leverage education policy to support students experiencing trauma, the difference between adversity and trauma must first be understood. Childhood adversity includes a wide range of experiences that can pose a serious threat to physical and/or psychological well‐being.^22^ These experiences are common among children and youth, affecting an estimated half of children under 18 in the United States.^23^ Further, the risk of childhood adversity varies by the child's age, race and ethnicity, special health care needs, and family income level, as well as many other risk factors. Notably, children living in poverty and children of color—particularly African American, American Indian, and Alaskan Native children—are at higher risk of experiencing adverse events due to the effects of inequitable distribution of resources and systematic racism and oppression.^24^, ^25^, ^26^ For example, American Indian and Alaska Native children and African American children have the highest rates of child maltreatment, at 15.2 per 1000 and 14.0 per 1000 in the United States, respectively.^27^

In population‐level studies, experiencing adversity in childhood has been repeatedly shown to increase children's risk for negative mental and physical health outcomes during childhood, adolescence, and later in life.^28^, ^29^, ^30^ However, an individual child's response to adversity can vary widely, from severe traumatic stress (eg, post‐traumatic stress disorder) to positive functioning under adverse conditions, or resilience.^31^, ^32^

Childhood adversity is often confused with trauma. While trauma is one possible reaction to adversity, children respond to adversity differently depending on the nature of the experience, its severity, frequency and duration, and the context of risk and protection in which it takes place. Thus, exposure to adversity does not necessarily result in trauma or long‐term negative outcomes. Most children who experience adversity return to prior levels of functioning with support from a sensitive, responsive caregiver. ^31^, ^32^

One subset of childhood adversity is adverse childhood experiences (ACEs). The seminal ACEs study included the following subset of childhood adversity: physical, sexual, and emotional abuse; emotional and physical neglect; having a mother who was treated violently; living with someone who was mentally ill; living with someone who abused alcohol or drugs; incarceration of a member of the household; divorce or separation of a parent. ^26^ This original list of ACEs is limited to exposure to adversity within the household, yet there are many common adversities—such as those that occur in the child's broader social environment—that were not included, such as poverty, homelessness, neighborhood violence, natural disasters and pandemics, and racism and discrimination, among others.^33^, ^34^ Today, some researchers and practitioners use the term ACEs to refer to an expanded list of adverse experiences, including those that a child experiences outside of the household, or experiences that were not on the original list of ACEs.^33^

Using the terms adversity, ACEs, and trauma interchangeably risks directing resources toward treating trauma where there may not be any, as well as overlooking children who have been traumatized but whose experience are not recognized under the term ACEs.^35^, ^36^, ^37^ Further, if schools or policy directives focus exclusively on ACEs, they may miss the broad range of childhood adversities that many students experience. The focus on ACEs is especially common as schools and policymakers turn toward ACEs screening tools to facilitate identification of students who need additional services.

### Screening for Adversity and Trauma

Multiple states have laws to encourage schools to screen for ACEs. Tennessee mandates that school boards maintain a policy compelling schools to assess students for ACEs prior to a suspension or expulsion.^18^ Pennsylvania established a school safety grant program that may be used to support ACEs screenings.^18^ California launched a statewide initiative to pay pediatricians and other health care providers to screen patients receiving Medi‐Cal for ACEs.^38^

Despite this momentum, there is limited evidence that universal screening for ACEs, adversity, and/or trauma in schools is an effective approach to supporting students, and one of the original ACEs study authors, Anda et al,^39^ recently warned against using ACEs‐based screening tools for screening individual children. Many screening tools in use or proposed for use by schools do not actually screen for trauma; instead, they screen for whether a child has experienced a subset of adversities, which may or may not have led to trauma.^40^ Such screening tools confound adversity and trauma and overlook a child's actual functioning following exposure. ACEs‐based and other tools that assess children's exposure to adversity or trauma typically take the form of a simple checklist of experiences that results in a single summary “score” of a number of adversities.^35^, ^36^, ^37^, ^39^ The checklist design gives equal weight to all forms of adversity, without accounting for the frequency, duration, or severity of the child's experience or the child's subsequent adaptation and functioning.^35^, ^36^, ^37^ Moreover, adversity screeners usually include a limited set of experiences that do not consider the potentially harmful impacts of children's environments beyond the home.

Further, the screening process itself can retraumatize children—forcing them or their parents to recount painful experiences without proper supports, especially if not conducted by well‐trained staff.^35^, ^39^ The results of the screening must also be handled carefully; for instance, by limiting who can access results and how findings are maintained in records, to avoid stigmatizing and further alienating the child and their family. In cases of child abuse and neglect and intimate partner violence, school staff must take care to engage families without exposing the child to greater harm^39^; as mandated reporters, staff must fulfill their obligations to report these instances to authorities. Further, the push to identify children experiencing trauma presumes that schools have appropriate in‐school services or systems available to refer children to community‐based services that can provide evidence‐based treatment.

### Training for School Staff on Trauma

Beyond screening, the most common provisions passed by states to address trauma encourage or require schools to train teachers and other school staff on trauma and trauma‐informed approaches. In 2017, 2 states (North Dakota and Wisconsin) mandated professional development for educators on the subject of trauma, while an additional 9 states (Washington, Oregon, Minnesota, Missouri, Texas, Virginia, Maryland, Connecticut, and Massachusetts) encouraged such training.^16^ Policies focused on training have continued to proliferate. In 2019 alone, Illinois, Maine, Washington, and Texas enacted new laws.^18^

As with screening, few studies have explored whether teacher training is effective at addressing trauma in schools.^19^ Recent reviews of trauma‐informed approaches in schools have all concluded that there is a dearth of evidence about what works for nonclinical staff. That is not to say that there is a lack of guidance on best practices; rather, very little of the guidance references strong evidence.^19^, ^41^ Previously, most research focused on educators examined efforts to build knowledge around the effects of trauma and to link students to more targeted interventions and supports.^41^ Recently, there have been more efforts to equip educators to take a more active role in supporting students experiencing trauma. However, few interventions delivered by educators have been rigorously evaluated, and there remain significant gaps with respect to how to effectively scale‐up efforts to equip educators to address trauma.^42^, ^43^

Consistent with teacher trainings on other topics, building educator awareness about trauma is likely critical to establishing a foundation for broader trauma‐informed approaches, but is unlikely to be successful without an infrastructure to reinforce and encourage the application of that knowledge.^44^ In practice, this means that training must be complemented with a range of practices, policies, and supports that apply SAMHSA's 4 trauma‐informed principles (recognize, realize, respond, avoid retraumatization). Unfortunately, because current state laws focus on training as the key requirement, schools may comply with these mandates without establishing these broader supports. Even for schools that do take steps to create trauma‐informed environments, evidence of effectiveness is still limited.^19^

### School‐Based Trauma‐Informed Frameworks and Programs

Recent examinations of trauma‐informed approaches published by professional associations and research centers,^19^ as well as national advocacy groups and state Departments of Education,^41^ reveal many lack clear references to empirical research. In a review of two decades of research on trauma‐informed practices in schools, Thomas et al^41^ note that while their review identified a number of frameworks with overlapping content, they were unable to identify a dominant framework. Rather, many resources highlighted connections between multi‐tiered systems of support (MTSS) such as positive behavior intervention in schools (PBIS) and trauma‐informed efforts, a trend that was highlighted in other recent reviews.^19^, ^45^, ^46^ These frameworks, which are similar to the public health model for prevention and intervention, call for whole‐school approaches (tier 1) as well as supportive and clinical intervention strategies (tiers 2 and 3) for those who are at risk for or experiencing trauma symptoms. The vast majority of states (40 as of 2019) encourage or require schools to implement MTSS, but few states do so with an explicit frame toward trauma.^47^ A recent pilot of efforts to implement trauma‐informed approaches within a PBIS framework—particularly tiers 1 and 2—suggests that there is potential for this framework to be useful, but additional research is needed.^48^

Whereas little is known about the impact of whole‐school trauma‐informed strategies, there is significant empirical support for clinical interventions for those identified as needing support in tiers 2 and 3 of a MTSS.^41^, ^45^ For example, cognitive behavioral interventions for trauma in schools and trauma‐focused cognitive behavioral therapy are well‐established with multiple rigorous evaluations.^19^ However, such targeted approaches may not be able to serve the needs of all students who are experiencing trauma. In particular, in their evaluation of a PBIS approach to trauma, von der Emse et al^48^ noted that schools with large proportions of students with unmet needs are likely to experience challenges as they work to implement tier 2 and tier 3 interventions; in many cases, more students need services than resources can provide.

Although MTSS can provide additional supports and services, they rarely address existing structures at school that may be contributing to or exacerbating trauma symptoms. For example, in reviewing existing trauma‐informed interventions, Gherardi et al^49^ found a general lack of recognition of the role that oppression and discrimination play when it comes to who experiences trauma and the ways in which children experiencing trauma are supported or further marginalized by schools. The authors noted that racism was not mentioned in any of the literature they reviewed as a “critical consideration in trauma‐sensitive schools.”^49^ ^(p 11)^ When race was mentioned, the authors found that it was generally to highlight the disproportionate exposure to trauma of historically marginalized groups of students—including students of color, students from immigrant families, and students who identify as lesbian, gay, bisexual, transgender, queer or questioning—without acknowledging and seeking to address the reasons why those groups of students experience more trauma than other groups. The authors call for a focus on dismantling structures that perpetuate trauma by partnering with marginalized communities to build meaningful relationships between schools and communities, while avoiding a deficit‐orientation. They additionally call for school staff to recognize the role that they have played as sources of trauma for many students, particularly students of color.

### Intersection of the WSCC and Trauma

State laws largely focus on helping schools recognize trauma, but do little to address how schools should incorporate the other components of SAMHSA's trauma‐informed principles, especially how schools should incorporate a knowledge of trauma into their approaches to addressing students' broader academic, physical, social, and emotional needs, and how schools should actively work to avoid retraumatization. Students who have experienced trauma require a sense of mental and physical safety and security to prevent further traumatization.^20^ To accomplish this, students need their basic needs met and strong connections to supportive adults and school environments.^50^, ^51^

The WSCC provides a natural framework with which to expand how states are guiding schools to address trauma. Within each of the WSCC's 10 domains, schools implement a variety of services, trainings, programs, and practices to support the development of the whole child. Many of these strategies are reinforced by state law and/or district policy.^16^, ^52^ Yet, these very strategies have the potential to either support students experiencing trauma or exacerbate trauma symptoms, depending on the approach.

For example, addressing student misbehavior is critical to ensuring students are able to focus on learning.^53^ It is normal for children who have experienced trauma to exhibit a range of internalizing and externalizing behaviors, including attention problems, impulsivity, anxiety, depression, aggression, and engaging in risky behaviors such as drug use.^54^ Thus, perceived misbehavior may in fact be a symptom of trauma. Schools vary significantly in their policies and procedures for addressing misbehavior, with many schools focusing on disciplining the behaviors—often through exclusionary mechanisms such as suspension or expulsion—rather than providing students support.^55^ Exclusionary approaches to discipline, such as suspension, are associated with detrimental academic and longer‐term outcomes for students^56^, ^57^ and may themselves be an adverse event that could exacerbate trauma.^49^

Schools also help ensure that all children have access to nutritious meals, with free and reduced‐price meals for low‐income children supported by the National School Lunch Program and the School Breakfast Program.^58^ For children with a history of childhood adversity or experiencing traumatic stress, it is particularly important for schools to provide consistent access to nutritious meals to promote physical and mental security. However, schools have struggled to identify financial solutions when children not eligible—or not registered—for free meals, do not have the funding to pay. To avoid accumulating meal debt, many schools have turned to shaming practices—including stamping children's arms, denying hot meals, and throwing away children's lunches—to compel parents and caregivers to repay meal debts.^59^ Such practices may be particularly harmful for children already experiencing trauma as it threatens their ability to meet basic needs and may expose them to further adversity.

Although schools serve a primary role in supporting student nutrition, many play a less direct role in helping students address other areas of basic needs such as physical and mental health care or housing. Ensuring students who are experiencing trauma have access to such services is critical for creating and maintaining their sense of security. Schools are often not the primary agency responsible for providing these services, but are a direct touchpoint for students and their families. Creating systems of “integrate student supports” or “wrap‐around services,” whereby schools develop deliberative partnerships with community agencies and organizations and serve as a hub for connecting families to services, can help directly address these needs.^60^

Yet, many schools provide only a minimal foundation for addressing these needs. Although all but 6 states require schools to provide a baseline of school‐based health services, these services are often far from comprehensive, and many states do not explicitly define what services must be offered.^16^ Only 19 states specifically address the creation of on‐campus health clinics or centers.^16^ Moreover, although 36 states address the availability of nurses on school campuses, only 1 (Vermont) explicitly requires schools to have a nurse on campus every day. Nationwide, only 40% of schools have a full‐time nurse, and 25% report having no nurse at all.^61^ Establishing these services require resources, but in some cases, state policy precludes schools from accessing available federal funding. For example, as of 2018, 33 states had policy barriers restricting use of Medicaid in schools, including for the provision of services delivered to all students (“free care”).^62^

Further, though schools are beginning to address student needs, they often lack policies and practices to support educators. As of September 2017, 39 states did not include any provisions related to employee wellness.^16^ For children experiencing trauma, redeveloping healthy relationships with teachers and other adults in school is a difficult but critical component of academic success and persistence and emotional well‐being.^51^ School staff may themselves have histories of trauma and may also experience secondary or vicarious trauma after hearing detailed stories of violence or maltreatment or witnessing a student's experience and its aftermath.^63^ Without strong mental health supports, maintaining strong relationships with students experiencing trauma is uniquely challenging for those experiencing vicarious trauma.^63^

### Summary

School discipline, school lunch shaming, integrated student supports, and employee wellness are just some of the issues that are driven by state policy and directly intersect with schools' ability to create a trauma‐informed environment. State policymakers must thus consider trauma not as a standalone issue, but as one that should be integrated throughout policies governing how schools address students' physical, social, and emotional needs. Simply requiring teacher training, especially when available trainings have only limited evidence, or mandating screening of students for ACEs or adversity, which carries several limitations and risks, cannot create the supportive environments students experiencing trauma need to thrive.

## IMPLICATIONS FOR SCHOOL HEALTH

Despite growing attention, policy efforts to address trauma in schools have so far been limited in their scope, rely on a poor evidence‐base, and require further consideration of the broader contexts and services that may either exacerbate or further ameliorate students' experiences of trauma. By leveraging the WSCC as a starting point, schools can create learning environments in which students' basic needs are met and where instructional practices and cultural norms create a safe and welcome space for all students.

Guidance developed by the first and second author,^17^ provides a framework for governors, legislators, state boards of education, and children's cabinets to develop policy to support comprehensive, coordinated initiatives, aligned with the WSCC, to build trauma‐informed school environments. Briefly, we highlight the framework's 7 principles for helping state policymakers take a more holistic lens in their approach to policymaking for schools.

First, policy should, “define a vision for school safety and support.” Here, policymakers are encouraged to build a coherent, coordinated vision for developing and maintaining environments where schools support the needs of the whole child so they are both safe and healthy, where child trauma and the creation of trauma‐sensitive learning environments are fully integrated into this vision. This principle builds upon the WSCC by setting the foundation for understanding that trauma is not a stand‐alone issue, but must be integrated in a coordinated way across the other areas of a school's efforts to create healthy learning environments.

Second, policy should “establish a taskforce to operationalize the state's vision.” There is no one‐size‐fits all approach to creating a safe and healthy learning environment. Consistent with implementation frameworks for the WSCC,^64^ decision‐making must be guided by the stakeholders for whom implementation will matter most. Thus, stakeholder groups should represent a cross‐section of school community members (administrators, teachers, students, parents), include state education and health officials, and be representative of the state's demographic diversity, with a deliberate effort to include underrepresented and marginalized communities. The first task of this body should be a systemic review—including current laws, policies, funding streams, agency capacity, and data collection and reporting—to identify where shifts, changes, and improvements are needed to help school communities achieve healthier learning environments.

Third, policy should “ensure a baseline of knowledge for all school staff on trauma.” As noted in our review, although the current evidence base for teacher training is limited, establishing a consistent understanding of the issue within schools is critical to changing practice.^44^ Thus, the framework acknowledges the work that several states have already done to include this element, but emphasizes that training cannot stand apart from the other components needed to create a supportive learning environment. Further, training must recognize that although trauma is widespread, it disproportionately affects black, Hispanic, American Indian, and Alaskan Native children and must acknowledge structural inequity and racism that may underly experiences of adversity, and the role schools play in furthering these inequities.^49^

Fourth, policy should “establish state supports for districts and schools.” State education and health agencies are well‐positioned to provide a range of direct supports—including technical assistance and guidance—to both schools and school districts. States can also aid schools by clarifying how communities can leverage both state and federal funding streams. Key federal programs include Student Support and Academic Enrichment grants (Title IV of the Every Student Succeeds Act), Medicaid (specifically, school‐based Medicaid programs), and the Maternal and Child Health Block Grant (Title V of the Social Security Act).

Fifth, policy should “reduce barriers to accessing health and safety supports.” Given that children experiencing trauma may need assistance meeting their basic needs, states should examine policy options to help schools provide these services, for instance, by expanding access to school‐based health centers and other integrated student supports.^60^ States should also identify policy barriers that limit the capacity of schools to provide such services, for example, by eliminating policy barriers related to using Medicaid in schools.

Sixth, policymakers should “reduce and replace policies that traumatize students.” As noted previously, schools can be sources of support for children experiencing trauma but can also create new traumatic or otherwise harmful experiences. In particular, policymakers should consider whether policies addressing elements of the WSCC need to be amended to better support students experiencing trauma (eg, school discipline, lunch shaming) or new policies need to be enacted to provide more direct support to student or educator needs (eg, employee wellness supports). States should be particularly careful to examine their policy environments to remove incentives that might encourage the use of harmful school‐based practices and enact provisions to inhibit their use.

Finally, policies should “establish funding mechanisms to support broad‐based local action planning.” Incentivizing schools to implement a more holistic approach to trauma often requires more than policies and technical assistance; many schools require direct financial support to hire necessary staff and otherwise implement the strategies developed under this framework. Thus, states should establish or repurpose funding opportunities to enable school communities to begin planning their transition toward an environment supportive of all children. Multiple states—including Massachusetts, Utah, Illinois, and Pennsylvania—have established grant programs to integrate trauma‐informed approaches or training into schools.^18^

State policymakers are beginning to recognize that childhood trauma has everyday implications for teaching and learning. The proliferation of state policy initiatives is a clear demonstration of their interest in generating solutions. Although well intended, current policy approaches fall short of what is needed to create trauma‐informed learning environments. By using the wider lens of the WSCC to identify the multiple areas of students' physical, social, and emotional wellbeing that intersect with trauma, states can better equip schools toward whole‐school reform.

### Human Subjects Approval Statement

Not applicable. This study did not involve any human subjects research.

### Conflict of Interest

The authors declare no conflict of interest.
